# Health risks associated with the pharmaceuticals in wastewater

**DOI:** 10.1186/s40199-017-0176-y

**Published:** 2017-04-12

**Authors:** Nasser Nassiri Koopaei, Mohammad Abdollahi

**Affiliations:** 1 Department of Toxicology and Pharmacology, Faculty of Pharmacy, Tehran University of Medical Sciences, Tehran, Iran; 2 Toxicology and Diseases Group, Pharmaceutical Sciences Research Center, Tehran University of Medical Sciences, Tehran, Iran

**Keywords:** Wastewater, Chemical and pharmaceutical residues, Toxicity, Regulatory framework, Public health risk

## Abstract

The overwhelming population growth in recent decades and water crisis along with limited and uneven geographical distribution of fresh water resources is a growing challenge for the economic and human development. Wastewater reclamation and use could be an alternative for intact water sources and a promising solution to water scarcity and unequal distribution. However, wastewater is a double-edged resource both as an accessible water source for food production and human usage and concurrently may carry uncharacterized content with unknown toxicological profile causing acute or long-term health risks. Pharmaceuticals, cosmeceuticals, nanomaterials and their chemical decomposition derivatives found in wastewater are not well known in many cases. Their unknown toxicity, teratogenicity and carcinogenicity profile associated with lack of monitoring and control measures impose a significant hazard risk on the public health. This paper reviews the evidence on the health risks associated with the wastewater use for irrigated food production and the imposed risk on the end consumers mainly from pharmaceutical industry and related research facilities. Then, we suggest an applied framework for planning and policy-making to mitigate the health risks and optimally employ reclaimed wastewater for human purposes.

## Introduction

### Wastewater and its importance: a pharmaceutical approach

The world population growth coincides with higher demand for resources. Limited and uneven geographical distribution of fresh water resources will be challenging for the growing population as a primary life requirement and for food production. Thus, the increasing demand, especially from the municipal regions will impose strict burden on the supply side for water resources. In such situation, wastewater recycling and reuse could be an alternative for intact water sources and a promising solution to water scarcity and unequal distribution [1, 2].

Wastewater has attracted remarkable attention during recent decades as a reliable source of water while there are certain concerns about its safety for human use. Wastewater may be defined as the out-coming used water flow from different resources like municipal, industrial plants and agriculture among which pharmaceutical industry is a major concern because of its efflux is of physiologically and toxicologically potent compounds [3–5]. Based on the applications, various wastewater types could be recognized such as urban, industrial or agricultural wastewater. Noteworthy, wastewater may be composed of one or more of the mentioned types. While new and research targeted compounds serve as novel future remedies for health problems, their unknown health effects, metabolic fate and accumulation pose drastic concern on the environmental system for their health impact assessment [6]. Wastewater could be collected and treated and then reused or discharged into a water source; otherwise, it may enter the water sources and carry all the unknown and biologically active pollutants directly or indirectly over to the human body [7–9].

Organized initiatives for wastewater use has been gaining significance in water resources security, green economy and population and municipal planning, but, health impacts imposed by the research medicinal agents developed in the urban areas and probably mixed with municipal wastewater could be inadequately or inappropriately treated [3]. While wastewater use emerges vital in issues pertaining to climate change, food security, safe drinking water supply and industrial applications, but agriculture sector is absorbing about 30% of the tertiary treated wastewater global use [10]. However, an unknown percentage should be added to account for a huge share of untreated or partially treated wastewater of the local pharmaceutical industry is used for agricultural purposes specifically in low-income and developing countries where access to medicine is top health system priority that overshadows some health hazards [11, 12].

## Wastewater contaminants and its human health risks

The growing and widespread use of wastewater in agriculture and other applications with or without inappropriate treatment present drastic public health risks that should be addressed. However, there are well developed and sophisticated technologies for the wastewater treatments, but they do not seem to be viable for low-income countries because of the high investment and technological barriers [3, 13].

As evident from the path that wastewater passes through homes, industries and other usage points, it may contain many pathogenic microorganisms, chemical and pharmaceutical residues health risks in case not very well mitigated. The chemical pollutants may include, but not limited to salts, metals, metalloids, residual drugs, organic compounds, endocrine disrupting compounds, and active residues of personal care products [14, 15]. Several parameters influence the type and severity of health risks incurred like the wastewater treatment extent, pollutant characteristics, human exposure and local risk factors. Infectious outbreaks remain the most critical concern in low-income countries, while the chemical and pharmaceutical pollution is the major health risk in developing and high-income countries [16, 17].

In this context, some developing countries face almost both types of health concerns. Most of the manufacturing pharmaceutical plants and academic/research institutes are located inside the city where they work on the synthesis of new nanomaterials, chemicals and pharmaceuticals. On the other hand, the untreated or partially treated wastewater that contains different chemicals and heavy metals may find its way to some local drinkable water wells. The issue gains more importance when we get to know that a comprehensive wastewater treatment plan does not exist for most of the cities and the untreated wastewater may be used for the irrigation purposes to grow vegetables for direct human use [18–23].

Wastewater may be employed in irrigation, groundwater sources, restoration, industries, environmental, potable and non-potable municipal use. In European countries, wastewater usage is mainly focused in agriculture, industry, municipal and mixed uses, but the low or untreated wastewater is much used for agricultural irrigation in low and middle income countries [3, 24].

The public health risk could be managed through plans, including wastewater pathogenic microorganisms and chemical pollutant removal and minimizing human exposure to wastewater.

Water quality approach is a major risk management approaches advocated by the World Health Organization (WHO) and Environmental Protection Agency (EPA) which implies that wastewater should be treated to the extent that meets certain water quality criteria to prevent any related risk especially health concerns [14]. This approach invites countries to consider their specific cultural, socioeconomic and environmental conditions in the optimized development and implementation of sustainable, economical and efficient risk management interventions [25]. However, it has been publicly accepted that the wastewater treatment level should match the purpose of reuse. Therefore, cost-effectiveness studies are very important to evaluate different risk management options for evidence based decision-making, intervention choice selections and resource allocation purposes mainly in low-income and developing countries [3].

The WHO pathogen reduction guidelines are based on an acceptable increased disease burden of one per million disability adjusted life year (DALY) loss per person per year and provides recommendations for pathogen reduction interventions:

- Health protection for wastewater-irrigated field workers against the risk of viral, bacterial and protozoan diseases that should meet a 3–4 log unit pathogen reduction through wastewater treatment.
- Health protection for the wastewater-irrigated food crops consumers against the risk of viral, bacterial and protozoan diseases that should meet a 6–7 log unit pathogen reduction through primarily wastewater treatment and then post-treatment human health-protection control interventions.
- Health protection for wastewater-irrigated field workers and wastewater-irrigated food crops consumers against the risk of helminthic diseases through decreasing the human intestinal nematode egg to less than one per liter [1, 14].

## Typology of contaminants in wastewater

### Microbial hazards

Raw wastewater may contain plenty of human microbial pathogens, including bacteria, helminthes, protozoa and viruses. Employment of untreated or partially treated wastewater vegetable irrigation may cause the transmission of infectious microorganisms to the products end users and farm workers. Epidemiological and clinical studies have revealed a significant risk of helminthiasis like ascariasis and giardiasis, enteropathogenic infections like cholera, typhoid, shigellosis, *H pylori* and *E coli* infection, Listeriosis, Salmonellosis, enterovirus infections like rotavirus and poliovirus among others which have been shown to directly correlate with inadequate wastewater treatment. [26, 27].

### Chemical hazards

Pharmaceutical industries involved in the manufacturing of finished dosage forms and drug development use water for different purposes. Pharmaceutical water could be categorized based on its use into general use water, manufacturing process water, and research and development water. The wastewater from the first category could be treated as municipal while the second one contains mostly the known product being manufactured. The third type contains different and unknown compounds, but in lower concentrations [28]. Moreover, active pharmaceutical ingredients (API) manufacturing plants that conduct large scale chemical synthesis processes to produce APIs may also release both the final API and the intermediates from the preliminary synthesis steps during the manufacturing process as the second type. However, the first and third categories remain the same for the API manufacturers [29, 30].

Wastewater carries three major chemical hazards classes with toxicological implications that includes acute and chronic toxicity, carcinogenicity, and reproductive, developmental, and neurotoxicity. It is postulated that carcinogenic and neurotoxic effects are not bound to thresholds. However, certain chemicals can produce different types of toxicities. Nevertheless, more than one toxic effect can be exerted by the same chemical substance [31]. Arsenic, 1,4-dioxane and vinyl chloride are sample carcinogens found in wastewater. However, the potential effects of low dose, but chronic exposure to pharmaceuticals and personal care products through wastewater are an evolving concern, especially because there exist no reliable long term toxicological studies for these compounds [32]. In addition, recent use of nanomaterials as nanopharmaceuticals requires close attention when it comes to mind that our current knowledge of nanotoxicology is pretty limited and the regulatory authorities experience a lag phase in enforcing control measures. Lack of objective and convenient measurement capabilities intensifies this concern in comparison to enteric infections [33, 34].

On the other hand, the scarcity of epidemiological data for numerous chemicals and pharmaceuticals mainly those used in low amounts, animal to human extrapolation necessity and inherent uncertainty and lack of handy chemical exposure monitoring technologies particularly for emerging entities like nanopharmaceuticals. Therefore, in many cases, qualitative measures are applied in wastewater reuse risk assessment rather than quantitative methodologies [32, 35].

Wide literature review shows that different classes of pharmaceuticals could be found in the wastewater including antibiotics (clarithromycin, ciprofloxacin, doxycyclin, erythromycin, methronidazole, norfloxacin, ofloxacin, roxithromycin, sulfamethoxazole, sulfapyridin, tetracyclin, trimethoprim), antiepileptics (carbamazepine), anticoagulants (warfarin), analgesics and anti-inflammatories (4-aminoantipyrine, antipyrin, codein, diclofenac, ibuprofen, indomethacine, ketoprofen, ketorolac, naproxen), lipid regulators (clofibric acid, fenofibric acid, bezafibrate, gemfibrozil, ezetimibe), steroidal compounds (esterogenic and androgenic drugs) beta-blockers (acebutolol, atenolol, celiprolol, metoprolol, propanolol, sotalol), diuretics (furosemide, hydrochlorothiazide), contrast media (amidotrizoic acid, diatrizoate, iotalamic acid, iopromide, iomeprol, iohexol, iopamidol), cosmetics (galaxolide, tonalide), psycho-stimulants (caffeine, paraxanthin), antidepressants (fluoxetin) [8, 36–45].

Several studies have shown that pharmaceuticals (e.g., carbamazepine, diclofenac, and gabapentin), artificial sweeteners (e.g., acesulfame), X-ray contrast media (e.g., iohexol and iopromide), and corrosion inhibitors (e.g., benzotriazole) are just partially removed in conventional wastewater treatment processes rendering their fate a remarkable importance because they are very simply transferred to the human body through plants [46]. Noteworthy, conventional and advanced wastewater treatment processes for removing pharmaceuticals from the water sources have comparable the efficiency. Conventional wastewater treatment processes including activated sludge (removal efficiency of 7-100%), biological filtration (6-71%), primary settling (3-45%), coagulation, filtration and settling (5-36%), sand filtration (0-99%) and advanced wastewater treatment processes ozonation (1-100%) (maybe coupled with ultrasound and sonocatalysis (23-45%), catalytic ozonation (9-100%)), UV irradiation (29%), photolysis (UV/hydrogen peroxide) (52-100%), dark and light Fenton (80-100%), UV/TiO_2_ (more than 95%), biomembrane (23-99%), microfiltration and reverse osmosis (91-100%), reverse osmosis (62-97%) and ultrasound (24-100%) mostly show a relatively wide range of efficiency that denotes locally optimized operational conditions based on the selected treatment process selected [46].

Riemenschneider and colleagues analyzed the 28 micropollutants and carbamazepine metabolites uptake in 10 different field-grown vegetable species from Jordan. Twelve micropollutants and six carbamazepine metabolites were detected in the samples with concentrations between 1.7 to 216 ng/g of dry plant weight. They also performed a primary health risk evaluation based on the concept of threshold of toxicological concern for nine compounds that did not reveal any significant for seven of the micropollutants, ciprofloxacin and 10,11-epoxycarbamazepine, however, more in depth toxicity profile data was required to run a comprehensive assessment [47, 48].

A monitoring study of 31 pharmaceuticals in the Lisbon's drinking water supply system, including the analysis of 250 samples of raw and drinking water indicated that 16 pharmaceutical compounds were quantified in the samples with concentrations ranging from 0.005 to 46 ng/L in raw water samples and 0.09 to 46 ng/L in drinking water samples analyzed. They also ran human health risk assessment and showed that based on the current toxicological data, exposure to trace levels of pharmaceuticals in drinking water poses very low risks to the consumer's health. However, there are not convincing data on the chronic effects of the pharmaceuticals and their chemical degradation and biotransformation products on human physiology [49].

The removal efficiency and fate of the antibiotic vancomycin in two pharmaceutical wastewater treatment plants in eastern China were studied. Vancomycin was found in all wastewater and sludge samples of both plants. The total treatment procedure removal efficiencies were at least to 99%. In spite of relatively very high removal efficiency, the results of the environmental risk assessment of vancomycin in the plants effluent revealed that still a significant environmental and health risks remain in the wastewater [50].

In another study, 64 pharmaceuticals and metabolites in source and finished water at 6 drinking and 2 industrial water purification plants in Japan were investigated. Thirty-seven substances were found in the source water samples with concentrations mostly lower than 50 ng/L except for 13 compounds. However, iopamidol level was higher than 1000 ng/L at most plants. Seven pharmaceutical compounds and 1 metabolite (amantadine, carbamazepine, diclofenac, epinastine, fenofibrate, ibuprofen, iopamidol, and oseltamivir acid) were removed through the treatment process however, plants using the more elaborate technology had a higher removal efficiency. They finally concluded that the residual compounds with the found levels in drinking water would not have significant pharmacological effects [51].

The uptake of pharmaceuticals in root crops irrigated with treated wastewater and potential health risks was assessed. It was shown that the nonionic compounds (carbamazepine, caffeine and lamotrigine) had significantly higher levels comparing to ionic ones (metoprolol, bezafibrate, clofibric acid, diclofenac, gemfibrozil, ibuprofen, ketoprofen, naproxen, sulfamethoxazole, sildenafil) in both plant species. However, the compounds’ concentrations were higher in leaves than the roots. For example, carbamazepine metabolites were basically detected in the leaves, where the metabolite 10,11-epoxycarbamazepine accumulated. They then employed the threshold of toxicological concern (TTC) approach to evaluate the associated health risk. Data showed that a child may absorb the lamotrigine TTC value through a daily intake of half a carrot (about 60 g). They drew the conclusion that some compounds accumulated in edible plant parts are at concentrations well above the TTC value that require close attention [52].

In another study, twenty-five pharmaceuticals in a Spanish hospital wastewater was studied. Sample analysis results revealed that twenty four compounds were present at levels ranging from 5 ng/L to 2 mg/L. Iomeprol had the highest concentration range of 424 to 2093 μg/L followed by acetaminophen (15–44 μg/L), furosemide (6–15 μg/L) and ofloxacin and trimethoprim (2–5 μg/L) while propyphenazone had the lowest concentration of 5 to 44 ng/L. They performed a screening type environmental risk assessment study on the released wastewater from the hospital and showed that eight investigated compounds (acetaminophen, diclofenac, ibuprofen, naproxen, clarithromycin, ofloxacin, trimethoprim, propranolol) could possibly pose significant risk to aquatic organisms. Considering the present dilution and degradation processes just ibuprofen incurred a moderate risk [53].

Human health risk for 26 pharmaceutical compounds (like acetaminophen, ciprofloxacin, gemfibrozil, warfarin) and some of their metabolites for which the US environmental monitoring data are available were investigated. The study showed that the ratios of measured environmental concentrations (MEC) to predicted no effect concentrations (PNEC) are mostly very low and consistent with predicted environmental concentrations (PEC) to PNEC ratio. The low ratios for the compounds revealed that the trace concentrations of the pharmaceutical compounds imposed no remarkable public health risk in the surface and drinking water [54].

## Agricultural implication of the contamination and the food cycle

Wastewater use in agriculture should be well thought because the benefits and risks of impromptu water reuse should be first justified. Challenges arise when wastewater irrigation may pose certain health and environmental impacts principally in low income countries where wastewater is not at least completely treated. The tradeoff between the economic valuation and health hazard risk remains an unresolved challenge. Progressively limited freshwater supplies coinciding with wastewater production and growing population and urbanization drives the public agencies and private firms in investing in reclaimed water use, which has a great impact on the local economy where wastewater is an available and cheap water source. Therefore, the complete biochemical assessment and economic valuation, especially of potential health risks and incurred costs through the food chain turns into a challenging matter [3, 55, 56].

## Nanomaterials and investigational contaminants in wastewater

Nanomaterials are generally emerging as a growing fraction of the material flows worldwide that contribute a great share to human life and well-being in different industries [57]. One certain application of nanomaterials happens in the pharmaceutical and medical fields that include nanopharmaceuticals, nanobiomaterials and nanotheranostics among others. As the extensive and widespread usage of these materials implies, the fate of the growing amount of used nanomaterials in the environment is critical [58] In 2010, silica, titania, alumina, and iron and zinc oxides formed the most abundant nanomaterials in the global market in terms of mass flow mainly applied in paint, optoelectronics, cosmeceuticals, energy, catalyst and environmental purposes. It has been estimated that in 2010, 63–91% of over 260,000–309,000 metric tons of global nanoparticles production released into the land that could find its way to fresh and wastewater sources [59–61].

Therefore, the vast presence and potential impacts of nanoparticles in the environment on the organisms and human health is a pervasive affair. These nanoparticles could partially aggregate, form temporary complexes with solid particles in suspensions, precipitate as sediments, accumulate in organisms and enter fresh water resources and then food materials with reportedly ecotoxicological effects on microorganisms, plants, invertebrates and fish. The fate of nanoparticles heavily depends on their physicochemical characteristics and the characteristics of the environmental system [62–64]. Though available toxicity data suggest low environmental risk of nanoparticles to the environment and human health, but our nanotoxicological knowledge of the potential effects of nanoparticles in the water resources for human health is limited [65, 66]. This problem requires consistent research to develop our understanding in different fields, including analytical quantification and physicochemical characterization, environmental fate and transport processes, ecotoxicology and nanotoxicology [59, 67, 68].

## Concluding Remarks: Regulatory framework and implications

Wastewater use is a growing fact that could cause significant levels of human health risk for the human beings and environmental deterioration, especially if inadequately treated. In this scope, pharmaceutical industries have an important role in the efflux of biologically and toxic agents. [69] To address this concern, country specific, locally adjusted and cost effective wastewater treatment and pathogen eradication measures should be implemented that requires industry cooperation in wastewater management policy supported by field research, feasibility study and the cost-effectiveness analysis [70]. WHO promotes a stepwise progressive approach to the efficient and safe wastewater use fed by methodical data [14]. Such programs can be monitored by improved health outcomes. However, the technological barrier is a major rate limiting factor in the expansion of safe wastewater use and becomes even worse when new chemical entities like pharmaceuticals and nanomaterials especially in countries where knowledge-based economic is growing but the monitoring measures do not develop concurrently [12].

Public health risk mitigation entails both treatment and post treatment approaches, although economic surveys should be performed to determine the cost utility analysis of wastewater treatment infrastructures [71]. Efficient health risk management requires a cooperative action plan devised by governmental authorities, scientific institutions and the pharmaceutical industry that enjoys scientific data and methodical approach. This approach involves most significantly influential actors in the wastewater production to use cycle and facilitates the wastewater management process [72]. The wastewater management should also consider the new and unknown sources of contaminants such as the research institutions as a source of investigational compounds whose acute and chronic health effects and possible toxicities are not appropriately characterized [73]. On the other hand, the financial institutions could be incentivized to support wastewater treatment facilities and infrastructure in an integrated multifaceted public health risk reduction plan typically presented in the WHO Guidelines [1, 14]. The challenging part of the approach remains inefficient public and private sectors mobilization and enforcement of law and regulations while scientific studies and technology availability play a critical role in the design and implementation of the policy package.

## Abbreviations

API: Active pharmaceutical ingredient
DALY: Disability adjusted life year
EPA: Environmental Protection Agency
LOQ: Limit of quantification
MEC: Measured environmental concentrations
PEC: Predicted environmental concentrations
PNEC: Predicted no effect concentrations
TTC: Threshold of toxicological concern
WHO: World Health Organization
